# The first mitochondrial genome of a South America parthenogenetic lizard (Squamata: Gymnophthalmidae)

**DOI:** 10.1080/23802359.2021.1951132

**Published:** 2021-07-15

**Authors:** Tuliana O. Brunes, Mariana L. Lyra, José A. Maldonado, Katia C. M. Pellegrino, Miguel Trefaut Rodrigues, Matthew K. Fujita

**Affiliations:** a Departamento de Zoologia, Instituto de Biociências, Universidade de São Paulo, São Paulo, São Paulo, Brazil; b Departamento de Biodiversidade e Centro de Aquicultura, Instituto de Biociências, Universidade Estadual Paulista (UNESP), Rio Claro, São Paulo, Brazil; c Amphibian and Reptile Diversity Research Center and Department of Biology, University of Texas at Arlington, Arlington, TX, USA; d Departamento de Ecologia e Biologia Evolutiva, Universidade Federal de São Paulo, Diadema, São Paulo, Brazil

**Keywords:** Amazonia, asexual, iTru, mitogenome, Reptilia

## Abstract

The mitogenome of the South American parthenogenetic lizard *Loxopholis percarinatum* Müller, 1923 (Squamata: Gymnophthalmidae), a uni-bisexual species complex, was recovered for three individuals from Rio Negro region, Amazonas, Brazil. The content and order of genes are typical for vertebrate mitochondrial genomes, and we recovered 13 protein-coding genes, 22 tRNA, and two rRNA (12S and 16S), in addition to partial fragments of the Control Region. A maximum likelihood phylogenetic analysis with mitogenomes of selected lizard families recovered *L. percarinatum* with *Iphisa elegans* Gray, 1851, the only other Gymnophthalmidae species available in GenBank.

Lizards are the vertebrate group assembling the largest number of true parthenogenetic species, in which female populations reproduce clonally with no contributions from males (Vitt and Caldwell 2014). *Loxopholis percarinatum* Müller, 1923, a leaf-litter Amazonian (Family Gymnophthalmidae), is one of these examples. This nominal diploid species is actually a species complex that also includes an unnamed triploid parthenogenetic species and an unnamed bisexual species directly involved in its origin (Brunes et al. 2019). The study of complete mitochondrial genomes (mitogenomes) of parthenogenetic species is of interest because they often show large tandem duplication along with the entire mitochondrial DNA sequence (e.g. *Heteronotia binoei* Gray, 1845; Fujita et al. 2007). Additionally, mitochondrial data have also been used to discriminate between diploid and triploid lineages of *L. percarinatum*, as they are morphologically cryptic and show high levels of genetic differentiation (Brunes et al. 2019; Pellegrino et al. 2005). This species complex is actually the oldest (Miocene) parthenogenetic lizard group in the world (Brunes et al. 2019).

Samples were collected in the Rio Negro river region, State of Amazonas, Brazil, and tissue samples were deposited at the MTR Tissue Collection from the University of São Paulo. Samples analyzed included two samples of *L. percarinatum* (MTR9940 – Terra firme, Estação Ecológica de Anavilhanas, lat: −2.4886, long: −60.8764; and MTR41460 –Boa Vista, lat: −0.3441, long: −65.4073), and one sample identified as *Loxopholis* cf. *percarinatum* (MTR40828 – Santa Helena, lat: -1.4161, long: −61.7767). Total DNA was extracted from muscle tissue stored in ethanol using a standard phenol-chloroform protocol (Sambrook and Russell 2006) and concentration was quantified using QUBIT 2.0 Fluorometer dsDNA BR Assay Kit (Life Technologies, Carlsbad, CA). Then, 100 ng was used to sequence the mitogenome following the mitochondrial sequencing protocol developed by Fujita’s laboratory, including a first step of nuclear genome digestion using exonuclease. The mitochondrial DNA was then amplified using strand-displacement amplification with Φ29 DNA polymerase (NEB, New England BioLabs). The library was constructed using the amplified mitogenome and Illumina universal primers containing custom nucleotide indexes (iTru indexing strategy; Glenn et al. 2019). The library was sequenced on the Illumina iSeq 100, producing 150 bp paired-end (PE) reads.

Raw sequence reads were trimmed for adapters and quality-checked using Trimmomatic v0.39 (Bolger et al. 2014) and all reads smaller than 45 bp were discarded. The mitogenomes were then assembled using interactive mapping with MITObim v1.9 (Hahn et al. 2013). The *Iphisa elegans* Gray, 1845 mitogenome (NC_048879, Vacher et al. 2020), a close Gymnophthalmidae lizard, was used as the initial seed and iterations were run until no additional reads could be incorporated into the assembly. The assembled sequences were checked in Geneious Prime, and the most complete sequence of *L. percarinatum* (MTR9940) was used as a seed in MITObim with an aim to improve coverage and/or assembly of some regions not mapped with *Iphisa* seed. Final annotation was done with the toolkit MitoZ (Meng et al. 2019).

We obtained linear sequences of 15,641–15,905 bp, containing the 13 protein-coding genes, 22 tRNA, the two ribosomal RNA sequences (12S and 16S), and small fragments of the Control Region. The gene order was the same described for *Iphisa elegans* and most other lizards. Overall base compositions varied from A (31.5–31.8%), C (27.4–28.2%), G (13.2–13.4%), and T (26.9–27.6%).

For phylogenetic analyses, we extracted the 13 protein-coding genes and the 12S and 16S rRNA genes from the three individuals of *L. percarinatum* and a subset of available squamate mitogenomes (Supplementary Table S1). We used only the protein-coding and rRNA genes to avoid ambiguous alignments. Next, each gene was aligned using MAFFT v7.45 (Katoh et al. 2002; Katoh and Standley 2013) plugin in Geneious Prime and the final alignment was composed by 15,889 bp. Then, we conducted a maximum likelihood phylogenetic analysis with RAxML v 8 (Stamatakis 2014) in Geneious Prime. We selected the rapid bootstrapping algorithm with 1.000 replicates and a partition scheme by genes (GTR GAMMA model). The consensus tree (Majority rule) resulted in a topology in which the relationships are consistent, in general, with those published (e.g. Goicoechea et al. 2016) and that corroborated the close relationship between the Gymnophthalmidae's species, *L. percarinatum* and *I. elegans* (Figure 1). Additionally, the tree also shows that *Loxopholis* cf. *percarinatum*, MTR40828 is genetically distant from the other two specimens (the uncorrected pairwise genetic distances for 16S gene between MTR40828 and other samples were 7.7–7.9%), probably because this individual might be bisexual or due to ploidy level differences, as we mentioned above (see Brunes et al. 2019 for further information). However, we lack karyotypic data for all three samples used herein to properly confirm the last (see MTR9940; Laguna et al. 2010). Finally, these new data represent the first mitogenomes of a parthenogenetic lizard from South America, a region that presents another five parthenogenetic species from two different families (Gymnophthalmidae and Teiidae; Vitt and Caldwell 2014), and the second of Gymnophthalmidae family. These data can be used as a reference for other lizard studies.

**Figure 1.**
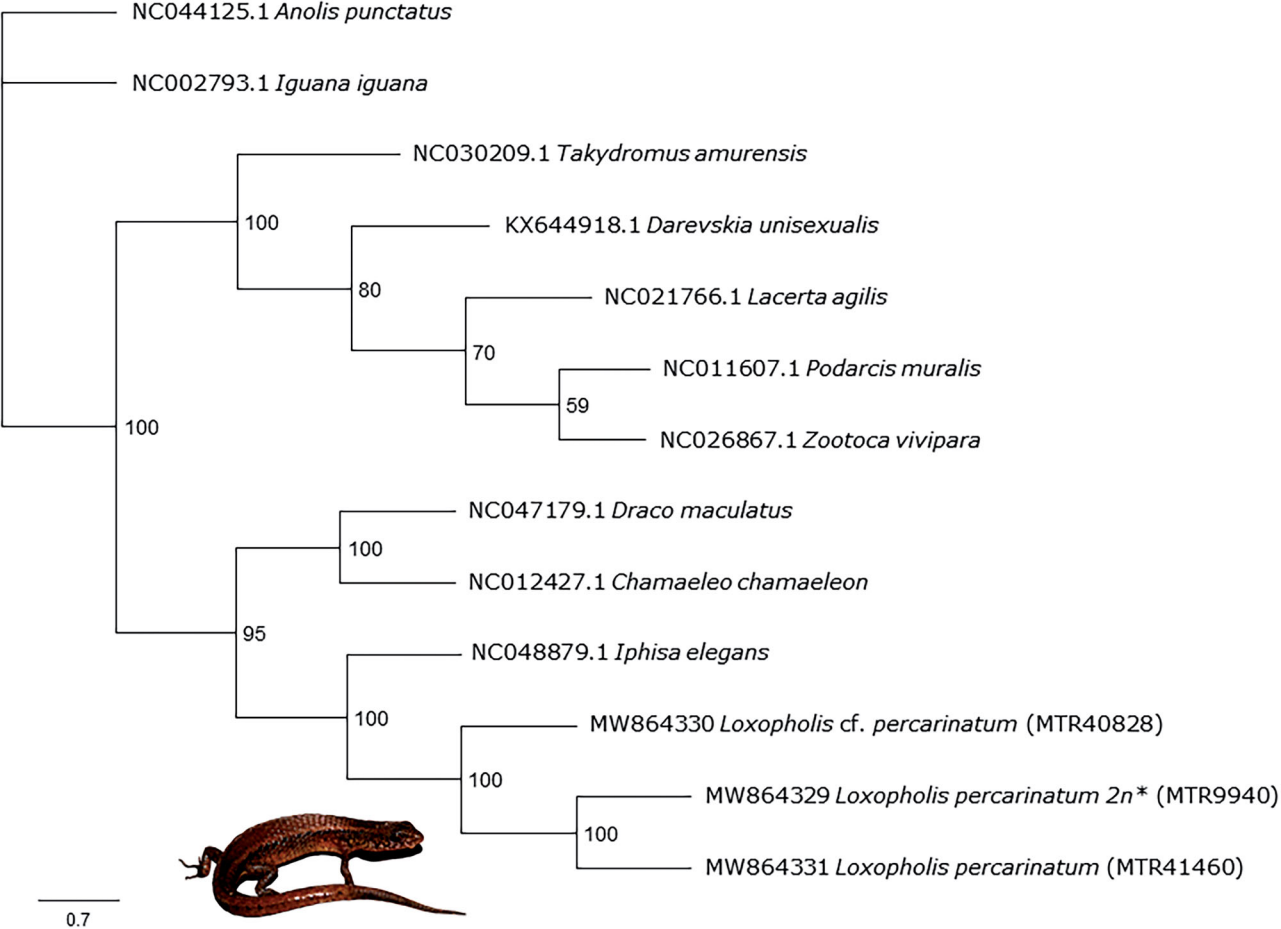
Maximum likelihood phylogenetic tree of thirteen squamate mitochondrial genomes that shows the placement of Loxopholis percarinatum complex. *Karyotype information from Laguna et al., (2010). The picture shows a specimen of L. percarinatum.

## Data Availability

The raw sequences data can be accessed in Sequence Read Archive, SRA (Accession number: PRJNA740025). The complete mitogenome sequences can be accessed in GenBank (Accession numbers: MW864329-31). Tissue samples are housed in the MTR collection from Universidade de São Paulo, state of São Paulo, Brazil, under the charge of Dr. Miguel T. Rodrigues (mturodri@usp.br). Specimens vouchers will be housed at Museu de Zoologia “MZUSP” from the same university, under the charge of the Curator of Amphibians and Reptiles Dr. Hussam El Dine Zaher (hzaher@usp.br), as soon as the access restrictions due Sars-Cov-2 (COVID19) pandemic are over. The Supplementary Table S1 is openly available in figshare at https://figshare.com/articles/dataset/Supplementary_Table_S1_docx/14374358.
